# CAR T-cell therapy in autoimmune diseases: a promising frontier on the horizon

**DOI:** 10.3389/fimmu.2025.1613878

**Published:** 2025-08-12

**Authors:** Dehong Wu, Zijun Y. Xu-Monette, Jia Zhou, Kepeng Yang, Xinchang Wang, Yongsheng Fan, Ken H. Young

**Affiliations:** ^1^ Division of Hematopathology, Department of Pathology, Duke University Medical Center, Durham, NC, United States; ^2^ Department of Rheumatology, The Second Affiliated Hospital of Zhejiang Chinese Medical University, Hangzhou, Zhejiang, China; ^3^ Institute of Basic Research in Clinical Medicine, College of Basic Medical Sciences, Zhejiang Chinese Medical University, Hangzhou, Zhejiang, China; ^4^ Duke Cancer Institute, Durham, NC, United States

**Keywords:** CAR T-cell therapy, autoimmune disease, autoreactive B cell targeting, Treg cell, personalized risk stratification

## Abstract

Although current treatments for autoimmune diseases can effectively control symptoms, they rarely lead to cures and often require lifelong use, accompanied by considerable adverse effects. This emphasizes the urgent need for more targeted therapies that offer long-term efficacy and curative potential. Chimeric antigen receptor (CAR) T-cell therapy presents a promising option by specifically targeting and eliminating autoreactive B cells, with the potential to reset the patient’s immune system and promote long-term immune balance. Originally developed for treating hematologic malignancies, where it has achieved remarkable success, recent studies have demonstrated substantial promise of CAR T-cell therapy, such as systemic lupus erythematosus (SLE) and myasthenia gravis. This article provides an overview of the current progress in CAR T-cell therapy for autoimmune diseases, focusing on five key approaches: CD19-targeted CAR T cells, CAR T cells targeting long-lived plasma cells, CAR T cells targeting specific autoantibodies, organ-specific CAR regulatory T cells (Treg cells), and mRNA-engineered CAR T cells. Additionally, this article discusses strategies for optimizing CAR T-cell therapy, including “off-the-shelf” allogeneic CAR T-cell therapy, combined CAR T-cell therapy, establishing timely consensus guidelines for their application in autoimmune diseases, and risk stratification strategies aimed at enhancing the personalization of treatments and minimizing adverse effects. While current research results are promising, further large-scale clinical trials and long-term follow-up are essential to thoroughly evaluate the safety and efficacy of CAR T-cell therapy in autoimmune diseases.

## 1 Introduction

Autoimmune diseases are a heterogeneous group of disorders caused by the misdirection of the immune system toward its host, and the presence of autoantibodies is a common feature of autoimmune diseases (1). There are nearly 100 different types of autoimmune diseases in humans, some of these are organ-specific, such as type 1 diabetes (T1D), while others are systemic, such as systemic lupus erythematosus (SLE) (2). Autoimmune diseases, previously considered rare, affect approximately 5%–8% of the world population according to epidemiological studies (3).

Given the limited understanding of the complex molecular mechanisms underlying autoimmune diseases, it is widely accepted that a combination of genetic predisposition and environmental factors disrupts immune tolerance (4–6), This disruption leads to the clonal expansion of autoreactive B and T cells, resulting in the production of autoantibodies that trigger autoimmune inflammation and the pathological attack on the body’s tissues. Current standard approaches offer no cure; rather, they focus on lifetime management. The primary approach for treating autoimmune diseases continues to involve controlling autoreactive T and B cells that attack host tissues, typically through the use of broad immunosuppressive agents such as glucocorticoids, methotrexate, and azathioprine (7–9). Recent advances have introduced a new paradigm of interventions, including biological agents and targeted small molecules, which more specifically inhibit detrimental immune cells and modulate specific inflammatory pathways. However, many patients with autoimmune diseases eventually relapse and may suffer from life-threatening complications (3). In summary, while current treatments can manage symptoms, they generally lack curative potential and are often associated with significant toxic side effects, some of which can be life-threatening. This highlights the urgent need for more optimized and curative therapies.

Chimeric antigen receptor (CAR) is a fusion protein consisting of an antigen recognition domain and a cell activation domain that can redirect T cell specificity, function, and metabolism (10). In oncology, CAR T cells have been used to accurately identify and eliminate cancer, showing remarkable and lasting efficacy in the treatment of certain cancers, particularly leukemia, lymphoma, and multiple myeloma (11–13). A decade-long follow-up study demonstrated that CAR T cells can persist in leukemia patients for up to ten years post-treatment, correlating with sustained disease remission and prolonged B-cell aplasia (14). Originally developed for cancer therapy, this approach mediates durable elimination of autoreactive B cells in autoimmune diseases, potentially eliminating the need for chronic immunosuppression. Importantly, it may also reestablish immune tolerance through secondary modulation of pathogenic T cell responses. Through this mechanism, CAR T-cell therapy, which targets autoreactive immune cells, is increasingly emerging as a potential curative approach for autoimmune diseases (15–18). It should be noted that CAR T-cell therapy is not intended to entirely replace existing treatment strategies. Instead, it is more appropriately positioned as a potential later-line option for patients with autoimmune diseases who exhibit inadequate responses to conventional therapies—including biological agents—or who are unable to tolerate their associated toxicities, particularly in cases of refractory or relapsing diseases. Recent preclinical studies, along with case reports and small-scale clinical trials, have yielded promising results in the treatment of autoimmune diseases using CAR T-cell therapy (19, 20). This review will focus on the clinical needs and applications, advantages and limitations, proposed solutions, and prospects of CAR T-cell therapy in the context of autoimmune diseases.

## 2 Classification and pathogenesis of autoimmune diseases

For clinical practitioners, autoimmune diseases are generally categorized into two main types: systemic and organ-specific. This classification is straightforward and practical, but its value in guiding treatment is limited, particularly as it does not reflect the underlying causes and immune mechanisms of the diseases. Therefore, a more precise classification based on specific pathogenic immune responses (such as abnormal T cell or B cell-mediated reactions) is beneficial for formulating personalized treatment plans. Characteristics of pathogenic immune responses include selective dysregulation of T cells or B cells, as well as dysregulated immune responses to specific self-antigens. For instance, in organ-specific diseases (such as T1D and thyroiditis), T cells play a crucial role, while in systemic diseases, such as SLE and rheumatoid arthritis (RA), B cells and their antibodies dominate. This classification method is beneficial in clinical treatment, as different immune mechanisms may require distinct therapeutic strategies (**Table 1**).

**Table 1:** Major organ-specific and systemic autoimmune diseases, targets, and mechanisms.

Autoimmune disease	Target organ	Autoantigen(s)	Primary mechanism of damage
Organ-specific			
T1D	Pancreatic β cells	Insulin, glutamic acid decarboxylase	T cells
Multiple sclerosis	Central nervous system	Myelin basic protein, myelin oligodendrocyte glycoprotein, proteolipid Protein	T cells
Pemphigus vulgaris	Skin and mucous membranes	Desmoglein 1, desmoglein 3	Antibody
Primary biliary cholangitis	Liver	Pyruvate dehydrogenase complex E2 component	T cells/Antibody
Myasthenia gravis	Neuromuscular junction	Acetylcholine receptor, muscle-specific Kinase	Antibody
Thyroiditis (autoimmune)	Thyroid	Thyroid peroxidase, thyroglobulin	T cells/Antibody
Autoimmune gastritis	Stomach	H+/K+ ATPase	T cells/Antibody
Autoimmune hepatitis	Liver	Cytochrome P450 enzymes	T cells/Antibody
Autoimmune oophoritis	Ovaries	Steroid-producing enzymes	T cells/Antibody
Autoimmune inner ear disease	Inner ear	Cochlear antigens, proteins in the inner ear structures	T cells/Antibody
Autoimmune orchitis	Testes	Sperm-specific proteins, testicular antigens	T cells/Antibody
Neuromyelitis optica spectrum disorder	Central nervous system	Aquaporin-4	Antibody
Idiopathic glomerulonephritis	Kidneys	Glomerular basement membrane, podocyte proteins	Antibody
Immune thrombocytopenia	Platelets and megakaryocytes	Glycoproteins on the surface of platelets, such as GPIIb/IIIa and GPIb/IX	Antibody
Systemic			
SLE	Skin, joints, kidneys, heart, lungs, central nervous system, blood cells, others	Nuclear antigens, others	Antibody
RA	Joints, lungs, heart, blood, others	Citrullinated proteins, rheumatoid factor	Antibody
Systemic sclerosis	Skin, lungs, gastrointestinal tract, heart, kidneys, others	Topoisomerase I (Scl-70), centromere proteins, RNA polymerase III	Antibody
Sjögren’s Syndrome	Exocrine glands, lungs, kidneys, liver,joints,others	Ro/SSA, La/SSB	Antibody
Polymyositis and dermatomyositis	Skeletal muscles, skin, lungs, heart, joints, others	Muscle antigens, aminoacyl-tRNA synthetases, other nuclear antigens	T cells/Antibody
Antiphospholipid syndrome	Blood vessels, pregnancy-related organs, brain, heart, Kidneys, lungs, others	β2-glycoprotein I, prothrombin	Antibody
Systemic vasculitis	Vessels, skin, kidneys, lungs, nervous system, heart, others	Proteinase 3 (PR3), myeloperoxidase (MPO), endothelial cell antigens	Antibody

T1D, type 1 diabetes; SLE, systemic lupus erythematosus; RA, rheumatoid arthritis.

**Figure 1:**
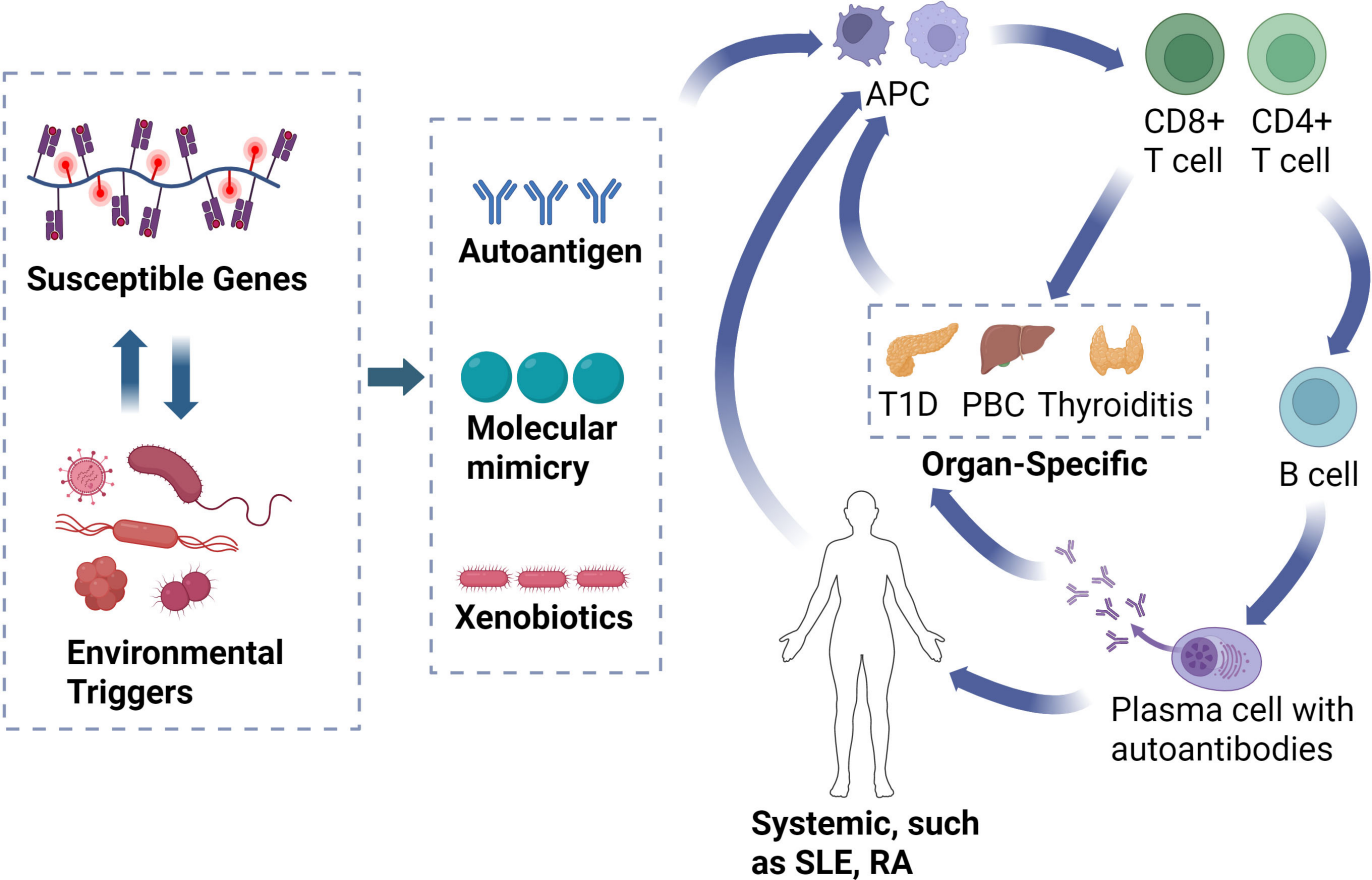
Illustration of the autoimmune cycle. The interaction between genetic susceptibility and environmental factors leads to the misrecognition of self-antigens. APCs present these self-antigens to T cells, initiating the immune response. Activated CD8^+^ T cells attack target organs, while CD4^+^ T cells activate autoimmune B cells to produce autoantibodies. These autoantibodies bind to antigens, forming immune complexes that deposit in organs, causing localized or systemic inflammation and damage. Immune complexes and inflammation continuously activate T and B cells, forming an autoimmune cycle that exacerbates tissue damage. APC, antigen-presenting cell; T1D, type 1 diabetes; PBC, primary biliary cholangitis; SLE, systemic lupus erythematosus; RA, rheumatoid arthritis.

The pathogenesis of autoimmune diseases involves a complex interplay between genetic susceptibility and environmental factors, with T cells and B cells collaboratively triggering and maintaining abnormal immune responses, forming an autoimmune cycle that ultimately leads to chronic inflammation and tissue damage (21) (**Figure 1**). Systemic autoimmune diseases, such as SLE and RA, exhibit widespread activation of the immune system, affecting multiple organs and tissues, and their pathogenic mechanisms involve self-antigen exposure and immune complex deposition (22). In these diseases, dendritic cells (DCs) present self-antigens to T cells (23), leading to the activation of T cells that secrete cytokines such as IL-21 and IFN-γ (24), which further activate B cells. Activated B cells undergo affinity maturation and differentiation into plasma cells, producing autoantibodies targeting a wide range of antigens (25). These autoantibodies bind to free molecules and antigens to form immune complexes, which can deposit in the vascular walls and tissues of multiple organs, triggering inflammation and damage (26). The deposition of immune complexes and the resultant local inflammatory responses establish an autoimmune cycle, continuously stimulating the activation of T cells and B cells, thereby exacerbating tissue damage and inflammation (27). Once this autoimmune cycle is initiated, if not interrupted by pharmacological intervention, the abnormal immune response persists, leading to the long-term maintenance and progression of autoimmune diseases (28).

In contrast, organ-specific autoimmune diseases, such as T1D and Graves’ disease, primarily target specific organs and involve immune responses against specific organ self-antigens. In these diseases, specific antigens, such as islet β-cell antigens or thyroid antigens, are presented to T cells by local antigen-presenting cells (APC), including DCs and macrophages. Activated T cells then attack the cells within the target organ by secreting specific cytokines like IL-17 and IFN-γ (29). For instance, in T1D, cytotoxic T cells directly attack islet β-cells, resulting in insufficient insulin secretion; in Graves’ disease, autoantibodies stimulate thyroid receptors, leading to hyperthyroidism (30, 31). These autoantibodies and local inflammatory responses create a specific immune cycle, which, unlike systemic diseases, is primarily confined to the affected organ (32).

In both systemic and organ-specific autoimmune diseases, the mechanism commonly involves the abnormal activation or dysregulation of T cells and B cells. This abnormal activation not only initiates the immune response but also sustains the pathological progression, forming an autoimmune cycle that makes the disease difficult to reverse (33, 34). Understanding this mechanism is crucial for developing effective treatment strategies. Although traditional therapies can alleviate symptoms and control disease activity, completely disrupting the autoimmune cycle remains a challenge (22, 35).

## 3 Limitations of current treatments for autoimmune diseases: highlighting the urgent need for novel therapeutic approaches

Current treatments for autoimmune diseases mainly include corticosteroids, non-steroidal anti-inflammatory drugs (NSAIDs), disease-modifying anti-inflammatory drugs (DMARDs), biologics, and targeted small molecules. Despite the variety of therapeutic options available, many patients are still predominantly treated with non-specific conventional treatments, with corticosteroids being among the most commonly used drugs. Corticosteroids can rapidly alleviate symptoms due to their powerful and broad anti-inflammatory and immunosuppressive effects. However, corticosteroids are a double-edged sword. Long-term or high-dose use can lead to serious complications, such as secondary infections, gastrointestinal ulcers, and femoral head necrosis, which can significantly impact patients’ quality of life and even be life-threatening (36–39). To reduce the cumulative dose of corticosteroids, DMARDs with steroid-sparing effects are often utilized. These include methotrexate, leflunomide, tacrolimus, azathioprine, mycophenolate mofetil, hydroxychloroquine, and cyclophosphamide (40–45). While DMARDs can help decrease the need for corticosteroids and lower the risk of disease relapse, patients in remission generally require lifelong low-dose maintenance therapy to prevent relapse. Furthermore, DMARDs do not offer targeted immunosuppressive effects; instead, they broadly inhibit various immune pathways, which can affect multiple cells in the body and increase the risk of secondary diseases, such as cancer (46).

Researchers are working to develop new treatments that are more effective, specific, and safer. Recently developed and clinically applied biological agents, such as various monoclonal antibodies and targeted small molecules like Janus kinase (JAK) inhibitors, have shown promise as treatment options for many autoimmune diseases, including SLE, RA, multiple sclerosis, myositis, psoriasis, atopic dermatitis, and pemphigus, without causing life-threatening adverse reactions (47, 48). The emergence of these novel drugs has alleviated some of the current treatment challenges and brought new hope to patients. For example, the anti-CD20 monoclonal antibody rituximab has demonstrated efficacy in several autoimmune diseases and has received marketing authorization for use in patients with SLE, granulomatosis with polyangiitis, microscopic polyangiitis, and pemphigus vulgaris (49). Belimumab, another biological agent used to treat SLE, works by inhibiting B cell survival and differentiation into antibody-producing plasma cells through the blockade of B-cell activating factor (BAFF) (50). Additionally, the targeted small molecule upadacitinib, a JAK inhibitor, blocks the phosphorylation of downstream effector proteins, thereby inhibiting cytokine signaling in key inflammatory pathways in immune cells. It has been approved for the treatment of RA and psoriatic arthritis (51).

Unfortunately, while these novel drugs can effectively attenuate the inflammatory process, they often require continuous treatment over many years or even a lifetime. Despite achieving remission, disease recurrence is common when immunosuppression is discontinued. This may be due to immune escape mechanisms that prevent the complete eradication of autoimmunity in patients with autoimmune diseases. Studies demonstrate that autoreactive B cells persist in specific tissue compartments after rituximab therapy, including SLE tonsils, systemic sclerosis skin lesions, and RA synovium (52–55). These findings suggest that, compared to circulating B cells, memory B cells in tissues are more resistant to depletion. Notably, these remaining tissue-resident B cells are thought to be responsible for disease relapse (53, 56).

## 4 The emergence of novel immunotherapies and comparison of emerging therapies

Current treatments for autoimmune diseases rely mainly on systemic immunosuppression, which generally reduces disease activity and can slow progression in many patients. However, this approach often requires ongoing medication. Prolonged use of immunosuppressive therapies can lead to side effects and complications, with some patients developing refractory disease states that are difficult to manage effectively.

Researchers have been striving to develop therapies capable of “resetting” the immune system, with the goal of profoundly recalibrating immune balance, terminating disease activity, and allowing patients to achieve long-term remission without the need for continued immunosuppressive therapy. In 1993, Marmont A. M. first proposed hematopoietic stem cell transplantation (HSCT) for severe, refractory lupus patients (57). This idea soon extended to severe autoimmune diseases (58, 59) and has been considered an alternative for SLE patients unresponsive to standard treatments (60). Multiple clinical trials suggest HSCT can induce long-term remission in various autoimmune diseases without maintenance therapy (61, 62). However, despite its advantages, the high rate of adverse effects, such as infections and secondary autoimmune diseases (63, 64), has limited HSCT’s use in autoimmune conditions like SLE (65). Further research is needed to assess the efficacy and safety of HSCT as a promising option for treating autoimmune diseases.

Dendritic cell (DC) therapy, a promising new immunotherapy that has made strides in cancer treatment (66), has recently been applied to autoimmune diseases (67). Through tolerogenic DCs, this approach induces immune tolerance in T cells against self-antigens, thereby suppressing autoreactive T cells in autoimmune disease management (68). Currently, DC therapy is in preclinical and early trials, demonstrating potential in inducing antigen-specific immune tolerance, thereby modulating T-cell responses and alleviating disease progression in RA (69), T1D (70) and multiple sclerosis (71). Despite this potential, challenges remain, including generating stable and functional DCs ex vivo and designing personalized DC vaccines tailored to diverse disease mechanisms. Further large-scale trials are required to validate its long-term efficacy and safety.

The application of natural killer (NK) cell therapy in autoimmune diseases remains in an early exploratory phase. NK cells can regulate the immune system by cytokine secretion and interacting with other immune cells (72, 73), modulating the activity of T cells, DCs, and macrophages, which impacts immune tolerance and inflammatory responses (72, 74). Consequently, NK cells show potential in suppressing autoimmune responses and restoring immune balance. Preclinical studies demonstrate that *in vitro* expansion and activation of NK cells effectively reduce inflammatory responses and improve clinical symptoms in the experimental autoimmune encephalomyelitis (EAE) model (75, 76). However, challenges persist, including precise control of NK cell activity to avoid damage to normal tissues, ensuring long-term stability, and optimizing applications across different autoimmune diseases. Despite these challenges, NK cell therapy’s immunomodulatory capacity and low toxicity present a promising therapeutic avenue for future treatment strategies.

Significant progress has been made in applying gene editing therapies to autoimmune diseases, primarily by modifying immune cell functions to inhibit pathological immune responses. The CRISPR/Cas9 gene editing technology, with its high efficiency, specificity, and flexibility, has been widely used to regulate T cells, B cells, and regulatory T cells (Treg cells), aiming to reduce their attack on self-tissues and restore immune tolerance. With technological advances, these approaches have shown promise in treating diseases like SLE, RA, and multiple sclerosis (77, 78). By targeting pathogenic genes or immune regulatory pathways, CRISPR/Cas9 holds promise for achieving personalized treatment and offers a novel strategy for precise intervention in autoimmune diseases. However, the long-term follow-up data on CRISPR/Cas9 gene editing technology remain limited, making it impossible to completely rule out potential long-term risks. The challenges of clinical translation and limitations of delivery systems further restrict the application of CRISPR/Cas9 in autoimmune diseases. Additionally, its high cost, ethical concerns, and the complexity of personalized treatment continue to limit widespread clinical application.

These therapies each have their advantages and limitations, and the most suitable option should be selected based on the patient’s disease characteristics, severity, and treatment goals. However, HSCT faces high risks and costs. Both DC and NK cell therapies are still in the early stages, and the long-term sustainability of their efficacy remains to be verified. Gene editing therapies, while precise, face ethical controversies and challenges related to personalized treatment. Recently, the development of engineered receptors has rapidly advanced CAR T-cell therapy (79). Initially applied to hematologic malignancies, CAR T-cell therapy has now been explored for autoimmune diseases. Early clinical applications have shown good safety and efficacy in diseases such as SLE and myasthenia gravis (80–82). Studies suggest that CAR T-cell therapy can target and eliminate pathogenic B cells, effectively control disease activity, and significantly reduce relapse risk, demonstrating potential for a cure (83). These initial successes have laid the foundation for expanding CAR T-cell therapy to a broader range of autoimmune diseases, gradually making it a preferred emerging therapy in the field. This review will focus on the prospects of CAR T-cell therapy in autoimmune diseases.

## 5 CAR T-cell therapies

### 5.1 Design and structure of CAR

A typical CAR comprises five functional domains: the antigen recognition domain, hinge region, transmembrane domain, co-stimulatory signaling domain, and T cell activation domain (84, 85). The antigen recognition domain is usually composed of a single-chain variable fragment (scFv) derived from the variable heavy (VH) and light (VL) chains of a monoclonal antibody, conferring high affinity and specificity toward the target antigen. Unlike conventional T cell receptors, the scFv enables direct recognition of cell surface antigens independent of antigen processing or MHC presentation, a feature particularly advantageous in autoimmune diseases with well-defined antigenic targets. The selection of CAR targets in autoimmune diseases must consider antigen specificity, expression patterns, and the risk of off-target effects. Numerous candidate targets have been proposed (**Table 2**), which provide a basis for the precise application of CAR T-cell therapy.The hinge region connects the scFv to the transmembrane domain, providing structural flexibility and facilitating optimal spatial orientation between the CAR and its cognate antigen. Common hinge elements include sequences from CD8α, IgG1, or IgG4, whose length and conformation can influence CAR surface expression, antigen-binding affinity, and potential cross-reactivity with non-target tissues. The transmembrane domain anchors the CAR to the T cell membrane and contributes to receptor stability and signal transmission. Frequently used transmembrane regions are derived from CD28, CD3ζ, or CD8α. The co-stimulatory signaling domain enhances T cell functionality and persistence. Second-generation CARs incorporate a single co-stimulatory signal, such as CD28 or 4-1BB, whereas third-generation CARs combine multiple co-stimulatory motifs to further amplify immune responses. The choice of co-stimulatory domain critically affects T cell metabolism, differentiation, and the cytokine release profile. Nearly all CAR constructs evaluated in clinical settings utilize the CD3ζ chain as the activation domain, which contains immunoreceptor tyrosine-based activation motifs (ITAMs) that initiate downstream signaling cascades leading to T cell activation, expansion, and cytotoxic function.

**Table 2:** Target antigens of CAR-based therapies in major autoimmune diseases.

Autoimmune disease	Target antigen	CAR strategy
SLE	CD19, BCMA	CAR-T
Multiple Sclerosis	CD19, CD20, XCR1, MOG	CAR-T / CAR-Treg
RA	CD19, CD20, BCMA	CAR-T
Systemic Sclerosis	CD19, BCMA	CAR-T
Dermatomyositis, Polymyositis	CD19	CAR-T
Sjögren’s syndrome	CD19, BCMA	CAR-T
Myasthenia gravis	CD19, BCMA, MuSK	CAR-T / CAAR-T
ANCA-associated vasculitis	CD19	CAR-T
Neuromyelitis optica spectrum disorders	CD19, BCMA	CAR-T
Autoimmune hemolytic anemia	CD19	CAR-T
PV	DSG3	CAAR-T
IBD / Crohn’s disease	IL-23R, FliC	CAR-Treg
Anti-NMDAR encephalitis	NMDAR	CAAR-T
T1D	Insulin, GAD65, other β-cell antigens	CAR-Treg

AIHA, autoimmune hemolytic anemia; ANCA, anti-neutrophil cytoplasmic antibody; BCMA, B cell maturation antigen; CAAR-T, chimeric autoantibody receptor T cell; CAR-T, chimeric antigen receptor T cell; CAR-Treg, chimeric antigen receptor regulatory T cell; DSG3, Desmoglein 3; FliC, Flagellin C; GAD65, Glutamic acid decarboxylase 65; IBD, inflammatory bowel disease; IL-23R, Interleukin-23 receptor; MOG, myelin oligodendrocyte glycoprotein; MuSK, muscle-specific kinase; NMDAR, N-methyl-D-aspartate receptor; PV, pemphigus vulgaris; RA, rheumatoid arthritis; SLE, systemic lupus erythematosus; T1D, type 1 diabetes; XCR1, X-C motif chemokine receptor 1.

With ongoing innovation in CAR design, their safety, efficacy, and durability are continuously optimized across diverse disease settings. In the context of autoimmune diseases, additional considerations include antigen specificity, minimization of off-target effects on healthy tissues, and fine-tuning of activation thresholds to mitigate immune-mediated tissue damage. To address these challenges, novel CAR architectures—such as inducible CARs (e.g., Tet-On systems) (86), bispecific CARs (87), and switchable CARs (88) are under active investigation. These strategies aim to achieve precise temporal and spatial control of CAR T cell activity, thereby enhancing therapeutic precision and safety.

### 5.2 CAR T cells targeting CD19

**Figure 2:**
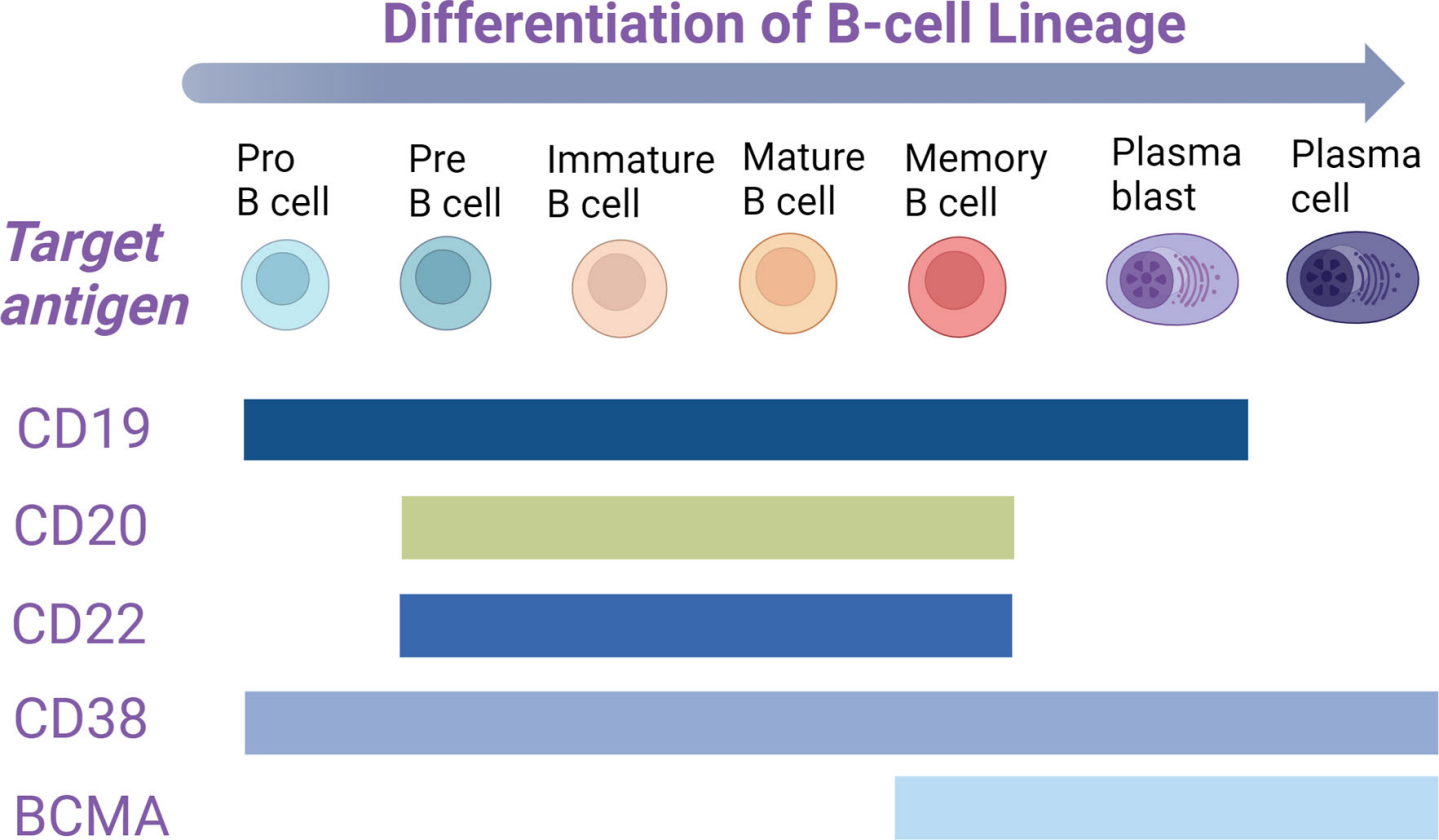
In the B-cell lineage, B cells at various developmental stages express distinct antigens on their surface. The expression stages of these markers are represented by colored rectangles. BCMA, B-cell maturation antigen.

Anti-CD19 CAR-T cell therapy can effectively eliminate tumor cells expressing CD19, resulting in significant clinical efficacy. Most patients experience progression-free survival for more than five years after treatment (89, 90). This success has sparked hope for the potential radical cure of autoimmune diseases (91). Given the diverse functions of T cells, such as tissue penetration, immunomodulatory effects, and cytotoxicity, CAR T-cell therapy holds promise for addressing various pathogenic mechanisms in autoimmune diseases. Therefore, identifying a suitable target antigen is crucial. CD19 is predominantly specific to the B cell lineage and is expressed across various stages of B cell differentiation, including in plasmablasts and a subset of plasma cells (92). Research has shown that although CD19 expression decreases in plasma cells, approximately 72% of CD38^+^CD138^+^ normal bone marrow plasma cells still express CD19 (93). However, terminally differentiated plasma cells, known as long-lived plasma cells (LLPCs), no longer express CD19 (94). These LLPCs reside in the bone marrow and are responsible for maintaining long-term antibody responses to previous viral infections and vaccinations. As expected, after anti-CD19 CAR T-cell therapy, most plasma cell compartments remain intact, and antibody titers from prior vaccinations remain stable. This preservation of humoral immunity and protective vaccine titers has been observed in both cancer patients and those with autoimmune diseases (95, 96), highlighting a potential immune-protective advantage of anti-CD19 CAR T-cell therapy. Moreover, anti-CD19 CAR T-cell therapy has demonstrated its efficacy in clearing B cells from tissues, even in the presence of large lymphoma masses (97, 98). In addition to CD19, other antigens such as CD20 and CD22 have also been explored as targets for CAR T-cell therapy in SLE and other autoimmune diseases. However, while the expression of these antigens overlaps with that of CD19, it is lower or absent in plasmablasts and plasma cells (**Figure 2**). Consequently, due to its broad and deep B cell depletion and its potential for preserving immune protection, anti-CD19 CAR T-cell therapy is emerging as a promising option for the treatment of autoimmune diseases.

In preclinical models of SLE, anti-CD19 CAR T-cell therapy has been shown to induce sustained depletion of CD19^+^ B cells, clear renal inflammation, and significantly extend the lifespan of model mice. These findings highlight the strong therapeutic and preventive potential of CAR T-cell therapy for SLE (99, 100). These milestone achievements in preclinical models provide robust support for the application of CAR T-cell therapy in autoimmune diseases and pave the way for subsequent clinical research and treatment. In 2021, CD19-directed CAR T cells were used for the first time to treat a 20-year-old woman with severe, treatment-refractory SLE (80). The patient exhibited various manifestations of lupus and responded poorly to multiple immunosuppressive agents, including belimumab and rituximab. Following the infusion of autologous anti-CD19 CAR T cells, rapid B cell depletion was observed, accompanied by a swift expansion of CAR T cells in the peripheral blood. Three months after the infusion, the patient achieved complete clinical remission, including the resolution of proteinuria and seroconversion of double-stranded DNA (dsDNA) autoantibodies. Moreover, all immunosuppressive medications, including glucocorticoids, were successfully discontinued, and there were no signs of disease relapse over the 18-month follow-up period. Importantly, the treatment was well tolerated, with no occurrence of cytokine release syndrome (CRS) or neurotoxicity (80).

Building on this promising case report where anti-CD19 CAR T cells successfully treated a patient with severe SLE, Mackensen et al. subsequently reported the use of anti-CD19 CAR T-cell therapy in five young adults diagnosed with SLE (81). Following CAR T cell infusion, the treated patients exhibited consistent expansion dynamics of CAR T cells, peaking around one-week post-infusion, which was accompanied by a marked reduction in disease activity. According to the DORIS criteria, all five patients achieved remission of SLE within three months, with peripheral B cells depleted to undetectable levels shortly after the infusion. Most pathogenic autoantibodies, including those specific to dsDNA, single-stranded DNA, and nuclear antigen Sm, were reduced to normal levels. The CAR T-cell therapy was well-tolerated, with three of the five patients experiencing low-grade CRS (Grade 1: fever), and no instances of neurotoxicity. Although B cell reconstitution was observed in all five patients, the reappearing B cells were predominantly of the IgM subtype, with a very low number of memory B cells. This suggests that the B cell compartment may have undergone a “reset,” potentially leading to the long-term depletion of autoreactive B cell clones. During subsequent long-term follow-up, no relapse of SLE was observed, even after the patients discontinued all SLE-related medications.

**Figure 3:**
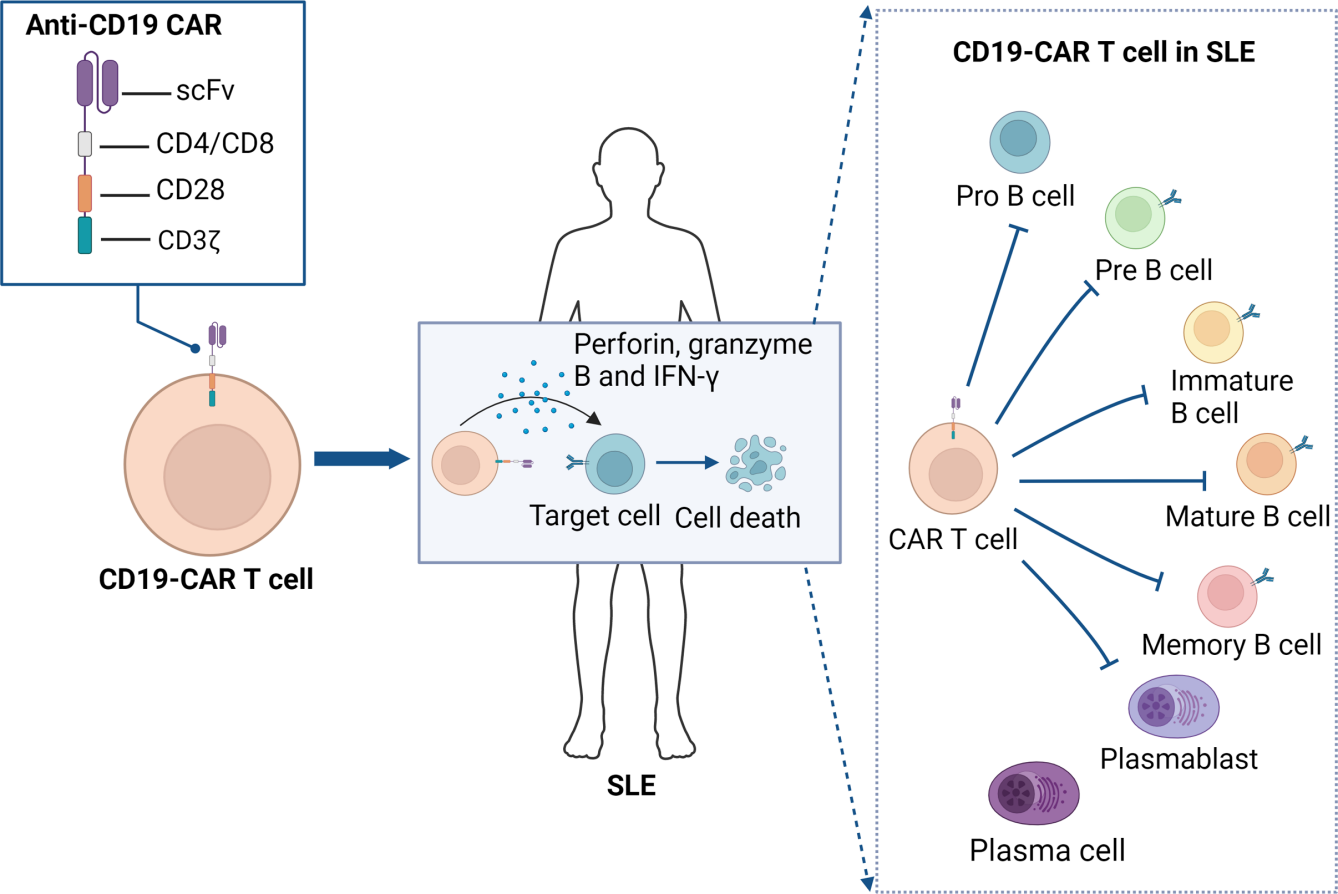
Anti-CD19 CAR T cell therapy for SLE. Anti-CD19 CAR T cells mediate immune modulation in SLE by targeting and eliminating CD19-expressing B cells. The scFv of CAR T cells specifically recognizes the CD19 antigen on the surface of B cells which, through the intracellular CD3ζ signaling domain and the CD28 co-stimulatory molecule, activates the CAR T cells, leading to the release of perforin, granzyme B, and IFN-γ, which induce B cell death. This mechanism inhibits the production of autoantibodies, thereby disrupting the autoimmune loop in SLE and ultimately achieving therapeutic goals. CAR, chimeric antigen receptor; scFv, single-chain variable fragment; SLE, systemic Lupus Erythematosus.

These encouraging research findings have led to a promising concept: if deep cellular depletion of autoreactive B cells can induce remission in SLE, it may also be effective for other autoimmune diseases. Following the success of SLE, clinical research has extended the use of anti-CD19 CAR T-cell therapy to other autoimmune conditions, such as systemic sclerosis (101–103), refractory immune-mediated necrotizing myopathy (103), refractory anti-synthetase syndrome (104, 105), and myasthenia gravis (96). These studies have all demonstrated significant efficacy and good tolerability, with no occurrences of CRS or other severe adverse events. While these results are promising, it is important to note that they are based on small cohorts of patients, and the full range of potential safety concerns will only become evident with extended long-term follow-up. Nonetheless, anti-CD19 CAR T-cell therapy is introducing a new treatment paradigm and is increasingly being considered a potentially curative approach for various autoimmune diseases, bringing new hope to patients (**Figure 3**).

### 5.3 Optimizing CAR T-cell therapy strategies by targeting long-lived plasma cells

B cell maturation antigen (BCMA), a member of the tumor necrosis factor superfamily, is predominantly expressed on plasmablasts (106), plasma cells (107), and some memory B cells (107) (**Figure 2**). These cells are responsible for producing autoantibodies that drive the development and progression of autoimmune diseases (108, 109). Anti-CD19 CAR T-cell therapy has shown promising results in treating autoimmune diseases such as SLE. The rapid decline in anti-dsDNA antibody levels observed in SLE patients undergoing anti-CD19 CAR T-cell therapy suggests that CD19^+^ plasmablasts and CD19^+^ plasma cells are the primary sources of these autoantibodies. However, unlike CD19^+^ cell populations, CD19- LLPCs primarily express BCMA and reside in the bone marrow (92, 110). These cells contribute to the production of autoreactive antibodies, such as anti-dsDNA antibodies (81), resist immunosuppressive treatments, and maintain kidney inflammation in mouse models of SLE (111, 112). Given the relatively short clinical follow-up time after anti-CD19 CAR T cell infusion, the possibility of disease relapse due to CD19- LLPCs evading CD19-targeted CAR T-cell therapy cannot be ruled out. Additionally, the severity of SLE and lupus nephritis has been correlated with increased expression of the BCMA surface antigen on LLPCs, making BCMA a promising therapeutic target (113).

A 5-year clinical outcome study on anti-BCMA CAR T-cell therapy in patients with relapsed/refractory multiple myeloma demonstrated significant efficacy and safety, providing important confidence and a reference point for the potential use of BCMA-targeted CAR T cells in the treatment of autoimmune diseases (114). Recently, BCMA-targeted CAR T cells were used to treat patients with myasthenia gravis, and both patients exhibited good safety profiles and sustained clinical improvement over 18 months, suggesting that the therapeutic efficacy may be linked to the reconstruction of the B-cell lineage accompanied by a sustained reduction in pathogenic autoantibodies (82). Additionally, in another clinical study targeting SLE, a bispecific approach simultaneously targeting BCMA and CD19 was employed. The results demonstrated that this strategy was safe and effective, leading to drug-free remission and the clearance of pathogenic autoantibodies (87). Furthermore, Qin et al. conducted an open-label, single-arm phase 1 clinical trial using anti-BCMA CAR T cells to treat neuromyelitis optica spectrum disorder (NMOSD). In this trial, 12 patients received anti-BCMA CAR T cells infusions, and the results indicated controllable safety and therapeutic potential (115). Moreover, Qin et al. performed a single-cell multi-omics analysis of blood and cerebrospinal fluid (CSF) samples from five NMOSD clinical trial participants treated with anti-BCMA CAR T cells. The findings revealed that anti-BCMA CAR T cells, enriched with chemotaxis gene programs, may be capable of crossing the blood-brain-cerebrospinal fluid barrier, eliminating abnormally expanded plasma cells in the CSF, and reducing the pro-inflammatory environment in the central nervous system in NMOSD (116). This suggests that anti-BCMA CAR T-cell therapy may have a natural advantage in treating autoimmune-related encephalopathies, including lupus encephalopathy.

CD38 is a multifunctional cell surface protein that plays a key role in inflammation and autoimmunity. It acts as an enzyme involved in nicotinamide adenine dinucleotide (NAD) consumption and intracellular signal transduction, while also functioning as an adhesion receptor (117). CD38 expression begins at the early stages of B cell development and increases progressively as B cells mature (118–120). It is highly expressed on plasmablasts, plasma cells, and memory B cells, with particularly elevated levels observed on LLPCs in the bone marrow (94) (**Figure 2**). The distinct expression pattern of CD38, coupled with its dual role in adhesion and ectoenzyme activity, has spurred the development of targeted CD38 antibodies (121). Daratumumab, the first antibody targeting CD38, has been shown to significantly reduce anti-dsDNA antibodies and vaccine-induced antibodies, lower antinuclear antibody titers, and markedly improve disease symptoms, suggesting its effectiveness in depleting LLPCs (122). However, as seen in the treatment of multiple myeloma, the response to daratumumab is often transient (123). SLE patients receiving daratumumab usually require continued immunosuppression or maintenance therapy with belimumab to prevent the regeneration of autoreactive LLPCs (122). Thus, CD38 represents a valuable target antigen for further development and clinical application.

### 5.4 Selective elimination of autoreactive B cells through targeting pathogenic autoantibody epitopes

**Figure 4:**
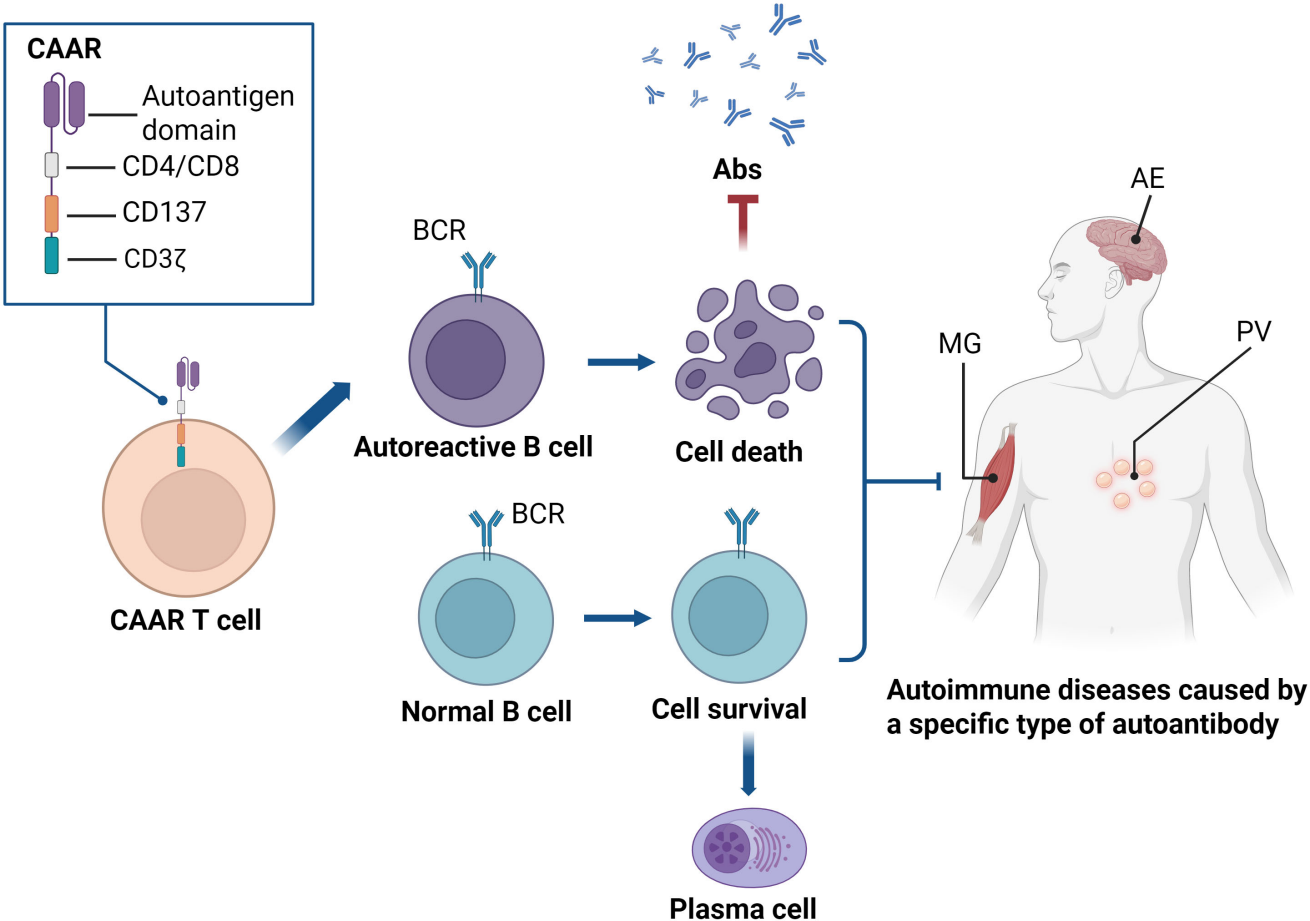
CAAR T cells are engineered by fusing a fragment of the autoantigen with the CD137 and CD3ζ signaling domains, aiming to specifically eliminate pathogenic B cells in autoantibody-mediated autoimmune diseases. Upon binding of the CAAR to the BCR on the B cell surface, CAAR T cells are activated, subsequently proliferate, persist, and selectively eliminate autoantigen-specific B cells *in vivo*, without affecting other B cell populations. This highly specific immune regulation provides a novel strategy for precision therapy in autoimmune diseases. CAAR, chimeric autoantibody receptor; Abs, autoantibodies; BCR, B cell receptor; MG, myasthenia gravis; AE, autoimmune encephalitis; PV, pemphigus vulgaris.

Despite the notable successes of CAR T-cell therapy in treating cancers and autoimmune diseases such as SLE, it is important to recognize that these strategies lead to the depletion of both autoreactive and healthy B cells. Following CAR T-cell infusion, patients experience B-cell depletion lasting weeks to months. During this period, they remain in a highly immunocompromised state. To address the issue of non-selective B cell depletion, therapeutic T cells have been engineered to express pathogenic autoantigen as the extracellular domains of chimeric immunoreceptors. This design allows for enhanced selectivity in targeting autoreactive B cells in autoantibody-mediated diseases with defined autoantigens, while preserving normal B cells (124, 125). These “reverse-engineered” T cells, known as chimeric autoantibody receptor (CAAR) T cells, represent a promising strategy for enhancing the specificity and safety of CAR T-cell therapy in autoimmune diseases (**Figure 4**).

Pemphigus vulgaris (PV) is a rare blistering skin disease caused by autoantibodies against desmoglein 1 and 3 (DSG1/3). Rituximab treatment significantly reduces anti-DSG antibodies and improves clinical symptoms. These findings underscore the critical role of B cells, particularly DSG-specific B cells, in PV pathogenesis (126–128). Preclinical studies have demonstrated that CAAR T cells engineered to express DSG3, fused to CD137-CD3ζ signaling domains, are capable of expanding, persisting, and selectively depleting DSG3-specific B cells *in vivo* (124, 129). These encouraging preclinical findings have guided the design of ongoing open-label clinical trials evaluating DSG3-CAAR T cells in patients with mucosal PV (NCT04422912) (**Table 3**). In the case of myasthenia gravis, anti-muscle-specific tyrosine kinase (MuSK) autoantibodies disrupt neuromuscular junction signaling, leading to muscle weakness (130–132). Preclinical studies in a myasthenia gravis model with circulating anti-MuSK autoantibodies have shown that MuSK-CAAR T cells can reduce anti-MuSK antibody titers without inducing widespread B cell depletion (133). A clinical study investigating MuSK-CAAR T cells for myasthenia gravis is currently underway and actively recruiting patients (NCT05451212) (**Table 3**).

**Table 3:** Ongoing clinical trials using different CAR T cells for the treatment of autoimmune diseases.

Clinical trial number	T cell type	CAR target(s)	Autoimmune disease	Phase	Sponsor
NCT06038474	CAR T cells	CD19	SLE	II	Cartesian Therapeutics
NCT06294236	CAR T cells	CD19	SLE, lupus nephritis, ANCA-associated vasculitis	I	Sana Biotechnology
NCT06465147	CAR T cells	CD19	SLE	I	Seattle Children's Hospital
NCT05798117	CAR T cells	CD19	SLE	I/II	Novartis Pharmaceuticals
NCT06121297	CAR T cells	CD19	SLE	I/II	Cabaletta Bio
NCT05869955	CAR T cells	CD19	SLE, idiopathic Inflammatory myopathy, systemic sclerosis	I	Juno Therapeutics, Inc., a Bristol-Myers Squibb Company
NCT06342960	CAR T cells	CD19	SLE, lupus nephritis	I/II	Kyverna Therapeutics
NCT06189157	CAR T cells	CD19	SLE	I/II	Miltenyi Biomedicine GmbH
NCT06347718	CAR T cells	CD19	SLE, systemic sclerosis, dermatomyositis, polymyositis	I/II	University of Erlangen-Nürnberg Medical School
NCT03030976	CAR T cells	CD19	SLE	I	Shanghai GeneChem Co., Ltd.
NCT05988216	CAR T cells	CD19	SLE	N/A	Bioray Laboratories
NCT06106906	CAR T cells	CD19	SLE	I/II	Wuhan Union Hospital, China
NCT06373991	CAR T cells	CD19	SLE	I	EdiGene Inc.
NCT06340490	CAR T cells	CD19	SLE	I	Guangdong Ruishun Biotech Co., Ltd
NCT06106893	CAR-γδ T cells	CD19	SLE	I/II	Wuhan Union Hospital, China
NCT06420154	CAR T cells	CD19	SLE, sjögren's syndrome, systemic sclerosis, inflammatory myopathy,	I	First Affiliated Hospital of Wenzhou Medical University
			ANCA-associated vasculitis, antiphospholipid syndrome		
NCT05859997	CAR T cells	CD19	SLE, sjögren's syndrome, systemic sclerosis, inflammatory myopathy,	N/A	Bioray Laboratories
			ANCA-associated vasculitis, antiphospholipid syndrome		
NCT06222853	CAR T cells	CD19	SLE in children	I	The Children's Hospital of Zhejiang University School of Medicine
NCT05765006	CAR T cells	CD19	SLE	I	Shanghai Ming Ju Biotechnology Co., Ltd.
NCT06310811	CAR T cells	CD19	SLE	N/A	Wuhan Union Hospital, China
NCT06549296	CAR T cells	CD19	SLE, systemic sclerosis, dermatomyositis, polymyositis, ANCA-associated vasculitis, idiopathic inflammatory myopathies, sjögren’s syndrome	Early I	Nanjing Bioheng Biotech Co., Ltd.
NCT06361745	CAR T cells	CD19	SLE, idiopathic inflammatory myopathies, systemic sclerosis, IgG4 related disease, primary sjögren 's syndrome	N/A	PersonGen BioTherapeutics (Suzhou) Co., Ltd.
NCT05930314	CAR T cells	CD19	SLE, lupus nephritis, immune thrombocytopenia		Peking Union Medical College Hospital
NCT04146051	CAR T cells	CD19	Generalized myasthenia gravis	II	Cartesian Therapeutics
NCT06359041	CAR T cells	CD19	Generalized myasthenia gravis	I/II	Cabaletta Bio
NCT05828225	CAR T cells	CD19	Myasthenia gravis	I	Zhejiang University
NCT06154252	CAR T cells	CD19	Idiopathic inflammatory myopathy, dermatomyositis, anti-synthetase syndrome, immune-mediated necrotizing myopathy	I/II	Cabaletta Bio
NCT06231368	CAR T cells	CD19	Autoimmune hemolytic anemia	I	Institute of Hematology & Blood Diseases Hospital, China
NCT06212154	CAR T cells	CD19	Autoimmune hemolytic anemia	I	Institute of Hematology & Blood Diseases Hospital, China
NCT06138132	CAR T cells	CD19	Multiple sclerosis	I	Stanford University
NCT06451159	CAR T cells	CD19	Multiple sclerosis	I	Bruce Cree
NCT06220201	CAR T cells	CD19	Multiple sclerosis	I	Juno Therapeutics, Inc., a Bristol-Myers Squibb Company
NCT05938725	CAR T cells	CD19	Lupus nephritis	I/II	Kyverna Therapeutics
NCT06298019	CAR T cells	CD19	Dermatomyositis	I	Stanford University
NCT06518876	CAR T cells	CD19	POEMS syndrome	I	Novatim Immune Therapeutics (Zhejiang) Co., Ltd.
NCT6688799	CAR T cells	CD19	Autoimmune diseases	I/II	Beijing GoBroad Hospital
NCT06056921	CAR T cells	CD19	SLE, sjögren’s syndrome, systemic scleroderma, dermatomyositis, ANCA-associated vasculitis	I	Chongqing Precision Biotech Co., Ltd
NCT06513429	CAR T cells	CD19	SLE	N/A	Peking University Third Hospital
NCT06585514	CAR T cells	CD19	SLE, lupus nephritis	I/II	Beijing GoBroad Hospital
NCT06193889	CAR T cells	CD19	Myasthenia gravis	II	Kyverna Therapeutics
NCT06544330	CAR T cells	CD19	SLE, lupus nephritis	I	Synthekine
NCT06588491	CAR T cells	CD19	Stiff-person syndrome	II	Kyverna Therapeutics
NCT06475495	CAR T cells	CD19	RA	I/II	Charite University, Berlin, Germany
NCT06333483	CAR T cells	CD19	SLE	I	Autolus Limited
NCT04561557	CAR T cells	BCMA	Neuromyelitis optica spectrum disorder, myasthenia gravis	I	Tongji Hospital
NCT06277427	CAR T cells	BCMA	Lupusnephritis, ANCA-associated vasculitis	N/A	Tongji Hospital
NCT06497387	CAR T cells	BCMA	Lupus nephritis, IgG4-related disease	I	Tongji Hospital
NCT06633042	CAR T cells	BCMA	Refractory AQP4 antibody positive neuromyelitis optica spectrum disease	I	Bioray Laboratories
NCT06428188	CAR T cells	BCMA/CD19	SLE, sjögren’s syndrome	I/II	Essen Biotech
NCT05858684	CAR T cells	BCMA/CD19	SLE	Early I	RenJi Hospital
NCT06350110	CAR T cells	BCMA/CD19	SLE, lupus nephritis, ANCA-associated vasculitis	I/II	Essen Biotech
NCT05474885	CAR T cells	BCMA/CD19	SLE	I	iCell Gene Therapeutics
NCT05846347	CAR T cells	BCMA/CD19	SLE	I	Zhejiang University
NCT06249438	CAR T cells	BCMA/CD19	SLE, immune-mediated necrotizing myopathy, neuromyelitis optica spectrum disorders, multiple sclerosis, myasthenia gravis	I	RenJi Hospital
NCT06503224	CAR T cells	BCMA/CD19	RA, SLE, primary sjögren’s syndrome, systemic sclerosis	N/A	The First Affiliated Hospital of University of Science and Technology of China
NCT06371040	CAR T cells	BCMA/CD19	Myasthenia gravis	I	Tang-Du Hospital
NCT06485232	CAR T cells	BCMA/CD19	Neuromyelitis optica spectrum disorders, generalized myasthenia gravis, multiple sclerosis, chronic inflammatory demyelinating polyradiculoneuropathy	I	Xuanwu Hospital, Beijing
NCT06419166	CAR T cells	BCMA/CD19	Generalized myasthenia gravis	I	Zhejiang University
NCT05263817	CAR T cells	BCMA/CD19	POEMS syndrome, amyloidosis, autoimmune hemolytic anemia, vasculitis	I	Zhejiang University
NCT05085431	CAR T cells	BCMA/CD19	Sjögren's syndrome	I	Zhejiang University
NCT05085418	CAR T cells	BCMA/CD19	Immune nephritis, lupus nephritis	I	Zhejiang University
NCT06497361	CAR T cells	BCMA/CD19	Lupus nephritis, IgG4-related disease	I	Tongji Hospital
NCT05030779	CAR T cells	BCMA/CD19	SLE	Early I	Zhejiang University
NCT06285279	CAR T cells	BCMA/CD19	Lupus nephritis, ANCA-associated vasculitis, membranous nephropathy-PLA2R induced, IgG4-related diseases	I	Nanjing University School of Medicine
NCT06733610	CAR T cells	BCMA/​CD19	Autoimmune hemolytic anemia	I	Institute of Hematology & Blood Diseases Hospital, China
NCT06785519	CAR T cells	BCMA/​CD19	Lupus nephritis	I	Zhejiang University
NCT06787989	CAR T cells	BCMA/​CD19	Refractory immune cytopenia	I	iCell Gene Therapeutics
NCT06340750	CAR T cells	BAFF	SLE	I	Luminary Therapeutics
NCT06279923	CAR T cells	CD19/BAFF	SLE, systemic sclerosis, dermatomyositis, immune nephritis, neuromyelitis optica	I	Zhejiang University
NCT06153095	CAR T cells	CD19/CD20	SLE, lupus nephritis	I/II	ImmPACT Bio
NCT06462144	CAR T cells	CD19/CD20	SLE, ANCA-associated sculitis, idiopathic inflammatory myopathy	I	The Affiliated Nanjing Drum Tower Hospital of Nanjing University Medical School
NCT06373081	CAR T cells	CD19/CD3E	SLE, sjögren’s syndrome, systemic sclerosis, inflammatory myopathy	N/A	Shanghai Changzheng Hospital
			ANCA-associated vasculitis, antiphospholipid syndrome		
NCT05239702	CAR T cells	CD7	Crohn Disease, ulcerative colitis, dermatomyositis, still Disease	I	Zhejiang University
NCT04422912	CAAR T/CAR T cells	DSG3/CD19	Pemphigus vulgaris	I	Cabaletta Bio
NCT05451212	CAAR T cells	MuSK	Myasthenia gravis	I	Cabaletta Bio
NCT05993611	CAR Treg cells	CD6	Chronic graft versus host disease, steroid refractory graft versus host disease	I	City of Hope Medical Center
NCT05234190	CAR Treg cells	HLA-A2	Liver transplant rejection, liver failure	I/II	Quell Therapeutics Limited
NCT04817774	CAR Treg cells	HLA-A2	Kidney transplant rejection, end stage renal disease	I/II	Sangamo Therapeutics

ANCA, anti-neutrophil cytoplasmic antibody; BAFF, B cell activating factor; BCMA, B cell maturation antigen; CAAR, chimeric autoantibody receptor; CAR, chimeric antigen receptor; N/A, not applicable; POEMS, polyneuropathy, organomegaly, endocrinopathy, monoclonal plasma cell disorder, skin changes; SLE, systemic lupus erythematosus; RA, rheumatoid arthritis.

N-methyl-D-aspartate receptor (NMDAR) encephalitis, the most common autoimmune encephalitis, is mediated by pathogenic autoantibodies targeting the NMDAR. To address this, NMDAR-specific chimeric autoantibody receptor (NMDAR-CAAR) T cells have been engineered to selectively target and eliminate B cells producing anti-NMDAR autoantibodies. Preclinical studies have demonstrated that NMDAR-CAAR T cells can effectively deplete anti-NMDAR B cell lines and maintain reduced autoantibody levels, all without inducing significant non-specific toxicity. These findings provide a foundation for future clinical trials (134). Moreover, in the context of immune thrombocytopenia (ITP), researchers have developed a novel chimeric autoantibody receptor targeting glycoprotein (GP) Ibα (GP Ibα-CAAR T cells). Compared to traditional CAR T-cell therapies, this approach enables the precise elimination of autoreactive B cells without causing broad B cell depletion. The efficacy and safety of GP Ibα-CAAR T cells have been validated in both *in vitro* and *in vivo* studies, highlighting their potential as a therapeutic option for patients with refractory or relapsed ITP (135).

### 5.5 Organ-specific CAR Treg therapy: targeted immune regulation approach

**Figure 5:**
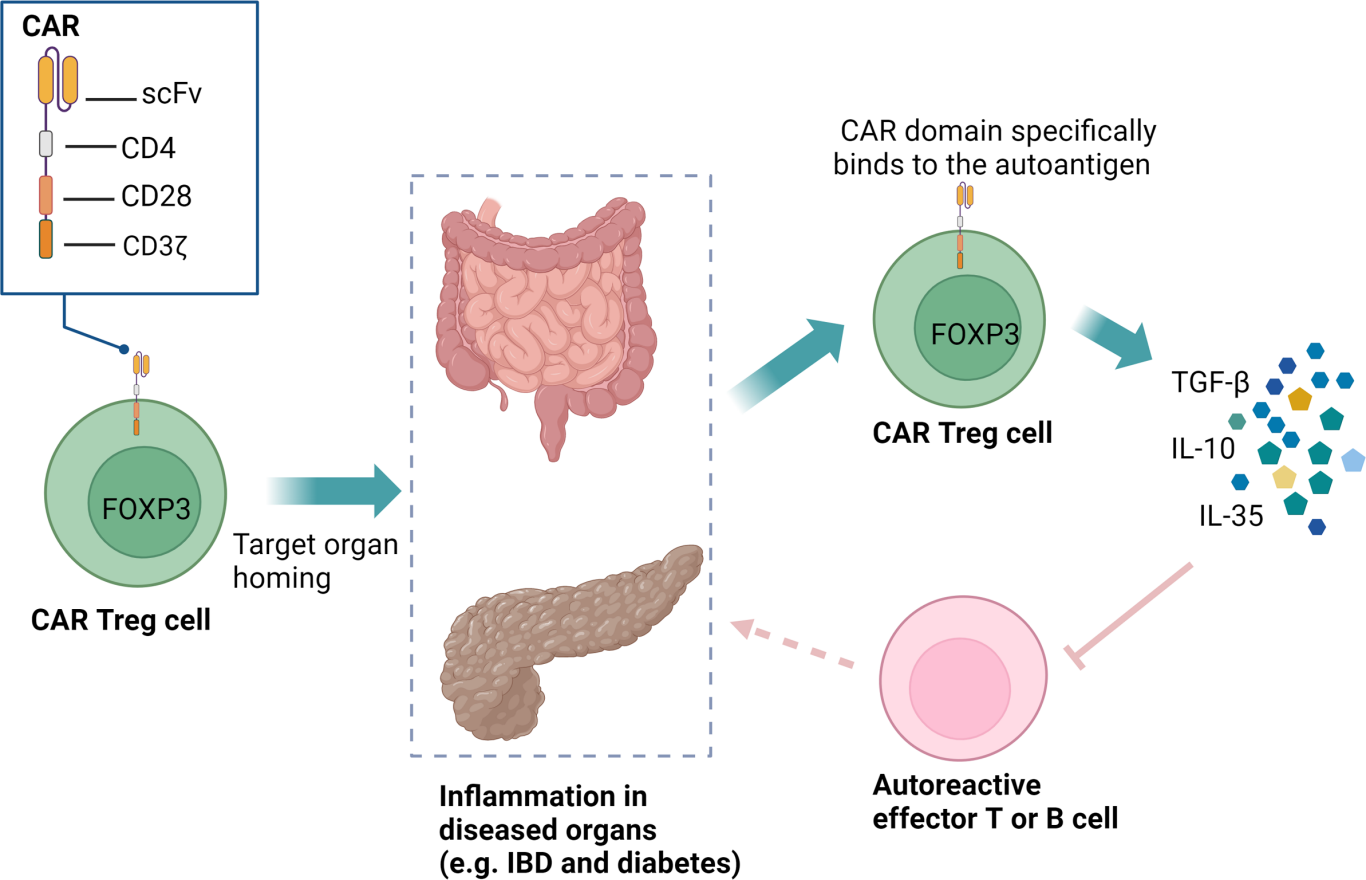
CAR Treg cells can specifically recognize organ-specific autoantigens and home to inflamed microenvironments. These CAR Treg cells, equipped with CD28 and CD3ζ signaling domains, can expand *in vivo*, persist long-term, and exert immunoregulatory functions through the secretion of TGF-β, IL-10, and IL-35. This precise targeting facilitates localized immunotherapy, effectively reducing the need for systemic immunosuppression and minimizing associated side effects. CAR, chimeric antigen receptor; Treg, regulatory T cells; IBD, inflammatory bowel disease; scFv, single-chain variable fragment; FOXP3, forkhead box P3.

Autoimmune diseases arise from a breakdown in immune tolerance to self-antigens, primarily due to inadequate suppression of aberrant immune responses by Treg cells (136). In recent years, significant efforts have been made to explore the therapeutic potential of Treg cells in treating autoimmune diseases (137, 138). However, thus far, these approaches have not yet achieved optimal therapeutic outcomes, primarily due to their inability to maintain long-term immune tolerance and the lack of clinical approval (139, 140). Interestingly, antigen-specific CAR Treg cells have shown the capacity to home to effector T cells (Teffs) and inflammatory sites in disease-relevant tissues, enabling targeted immune suppression in affected tissues or organs (140–142) (**Figure 5**). This targeted approach has the potential to reduce systemic immunosuppression, thereby minimizing associated side effects. In contrast to conventional CAR T-cell therapies that eliminate autoreactive cells, CAR Treg modulate immune responses by suppressing (but not killing) pathogenic lymphocytes, thereby promoting long-term tolerance (143, 144). Additionally, CAR Treg therapy offers another distinct advantage: stable Treg cells do not produce pro-inflammatory cytokines, which significantly lowers the risk of CRS typically associated with CAR therapies (145, 146).

CAR Treg cells have the potential to selectively target disease-associated tissue antigens affected by self-reactive immune responses, thereby enabling localized and organ-specific therapeutic strategies. This makes them highly promising for treating organ-specific autoimmune diseases such as inflammatory bowel disease (IBD) (147), multiple sclerosis (148), and T1D (149). Furthermore, CAR Treg cells are being explored as a strategy to induce immune tolerance and prevent organ rejection in transplantation (150, 151). A novel CAR targeting the flagellin protein derived from Escherichia coli H18 (FliC) has been developed. FliC-CAR Treg cells can recognize and respond to soluble flagellin independently of toll-like receptor 5, promoting intestinal homing in a humanized mouse model and exhibiting potent immunosuppressive effects. These findings support the clinical development of CAR Treg cells for IBD treatment and reveal the potential of CARs targeting microbial antigens (152). A survey by Vent-Schmidt et al. showed that most IBD patients would be willing to try CAR Treg therapy, highlighting its clinical promise (153). In a preclinical study on the treatment of multiple sclerosis, the transfer of X-C motif chemokine receptor 1 (XCR1)-specific CAR Treg cells into EAE, an animal model of multiple sclerosis, successfully inhibited T helper 1 cells (Th1)-driven EAE (154). Tenspolde and colleagues used CAR technology to redirect T cell specificity to insulin and reprogram Teffs into Treg cells through Forkhead box P3 (FOXP3) transduction. The data indicated that these insulin-specific CAR Treg cells were functionally stable, suppressive, and long-lasting *in vivo*, demonstrating the potential for restoring immune tolerance in T1D (149). In transplant medicine, the primary goal is to establish immune tolerance to prevent organ rejection without lifelong pharmacological immunosuppression. CAR technology has been used to direct human Treg cells toward specific HLA class I molecules. Drawing upon previous studies (155–158), Proics and colleagues further developed a lentiviral vector encoding a humanized single-chain variable fragment (scFv) specific to the HLA-A02 antigen (TX200) for clinical applications and optimized the isolation strategy for autologous initial (CD45RA^+^) human Treg cells (TR101). The resulting HLA-A02 CAR Treg cells were named TX200-TR101. A first-in-human trial is currently planned, marking the first clinical trial investigating CAR Treg cells (151).

### 5.6 mRNA-based CAR T-cell therapy: a novel treatment with transient *in vivo* expression and rapid therapeutic effects

The B cell burden in autoimmune diseases is generally much lower than in cancer patients, allowing CAR T cells to quickly eliminate cells carrying the target antigen *in vivo*. The loss of the target antigen may trigger early contraction of the CAR T cell population. Deep B cell depletion is sufficient to eliminate disease activity and reset the B cell system (124, 159). The long-term persistence of functional CAR T cells in autoimmune diseases seems to have limited value. Prolonged B cell depletion is not beneficial in autoimmune diseases because B cells contribute to humoral protection and are involved in immune homeostasis (160). Patients with permanent B cell depletion exhibit an increased risk of infections and poorer outcomes, such as in the case of COVID-19 infection (161). Traditional CAR T cells use lentiviral or gamma-retroviral vectors for permanent genetic modification, which poses risks of genotoxicity and regulatory challenges, and may also result in the lifelong persistence of CAR T cells (162, 163).

Currently, mRNA CAR T-cell therapy demonstrates tremendous potential in overcoming the limitations of traditional CAR T treatments. This therapy combines mRNA gene modification technology with CAR T immunotherapy, achieving cell transformation through *in vivo* targeting and *in situ* reprogramming of T cells, thus avoiding the complex and time-consuming ex vivo manufacturing process typical of traditional CAR T-cell therapy. By utilizing lipid nanoparticles (LNPs) as efficient delivery vectors, mRNA CAR T cells can precisely deliver mRNA encoding the CAR to T cells, enabling them to rapidly synthesize functional CAR molecules and initiate targeted immune responses (164). However, due to the transient nature of mRNA, CAR expression and immune activity gradually decline within days as the mRNA degrades (165). Thanks to its transient expression mechanism and high flexibility, mRNA CAR T-cell therapy offers the unique advantage of repeatable administration based on treatment needs (166, 167). By precisely adjusting mRNA dosage and administration frequency, the intensity and duration of the treatment can be dynamically controlled to better adapt to changes in the patient’s condition, such as disease relapse or heterogeneity of target antigen expression. This ability for repeated dosing provides significant flexibility for personalized treatment and long-term management. Compared to traditional CAR T-cell therapy, mRNA CAR T-cell therapy effectively reduces long-term toxicity risks due to its transient expression characteristics. The non-persistent expression avoids the issue of CAR genes remaining in T cells for prolonged periods, thereby reducing the occurrence of side effects such as CRS, neurotoxicity, and organ damage. The transient CAR expression may also reduce the risk of antigen escape or tumor resistance. More importantly, because mRNA does not integrate into the T cell genome, its non-integrating nature significantly reduces the potential risk of tumorigenesis. Additionally, the high adjustability of mRNA CAR T-cell therapy makes the treatment regimen more flexible, particularly for high-risk patients or those who need to avoid long-term immunosuppression (168).

In mouse models, injection of CD3-targeted LNPs carrying anti-CD19 CAR mRNA successfully induced CAR expression in T cells. These nanoparticles transduced CAR T cells, which significantly enhanced therapeutic efficacy against B-cell leukemia, as evidenced by a marked reduction in tumor burden and a notable extension of survival in the mice (169). Rurik et al. showed that T cell-targeting LNPs delivered mRNA encoding a CAR against activated fibroblasts in mice, generating functional CAR T cells. In a mouse model of heart failure, a single injection of CD5-targeted LNPs efficiently delivered the modified mRNA to T cells, producing transient yet effective CAR T cells. These CAR T cells accumulated in the spleen, exhibited phagocytic activity, maintained target antigen recognition, and ultimately improved cardiac function while attenuating fibrosis (170). These studies suggest that mRNA CAR T technology, with its transient and controllable immune cell functionality, shows unique potential in modulating immune balance and suppressing aberrant immune responses, indicating its promising applications in the treatment of autoimmune diseases. The first clinical trial of mRNA CAR T-cell therapy in autoimmune diseases targeted myasthenia gravis. The MG-001 clinical trial is a prospective, multi-center, open-label phase 1b/2a study designed to evaluate the efficacy and safety of anti-BCMA mRNA CAR T-cell therapy in treating myasthenia gravis. The trial treated a total of 14 patients using electroporated mRNA-based CAR T cells without lymphocyte-depleting chemotherapy. The follow-up period ranged from 3 to 9 months. The results indicated that the treatment was safe, well-tolerated, and significantly reduced the clinical severity of myasthenia gravis, highlighting the clinical potential of mRNA CAR T-cell therapy for treating myasthenia gravis and other autoimmune diseases (171).

## 6 CAR T-cell therapy toxicities: CRS, neurotoxicity, and management strategies

While CAR T-cell therapy shows great therapeutic promise, it is also associated with serious toxicities, particularly CRS and immune effector cell-associated neurotoxicity syndrome (ICANS), which have become major safety challenges in clinical applications. CRS is the most common adverse event following CAR T cell infusion. It is triggered by the robust activation of CAR T cells and other immune effector cells, leading to a massive release of inflammatory cytokines such as IL-6, IFN-γ, and TNF-α (172). Clinical manifestations range from fever, hypotension, and hypoxia to severe multi-organ dysfunction. The American Society for Transplantation and Cellular Therapy (ASTCT) has proposed a grading system to standardize the assessment of CRS. The risk and severity of CRS are influenced by tumor burden, CAR construct design, and T cell dose. ICANS is another severe toxicity, which often occurs after or concurrently with CRS but can also develop independently. Its pathogenesis involves cytokine-mediated endothelial activation, increased blood-brain barrier permeability, and neuroinflammation (172). Patients may present with confusion, language impairment, seizures, or even cerebral edema. A hallmark of ICANS is the rapid onset of neurological symptoms, typically within one week after infusion.

Currently, tocilizumab, an IL-6 receptor antagonist, is the first-line treatment for CRS and can rapidly alleviate symptoms without compromising the efficacy of CAR T cells. Glucocorticoids are used for moderate to severe CRS and for ICANS, particularly when tocilizumab proves ineffective. Due to its limited ability to cross the blood-brain barrier, tocilizumab is less effective for ICANS, making glucocorticoids the mainstay of treatment. Current preventive strategies primarily include preemptive IL-6 blockade, individualized adjustment of CAR T cell dosage, and the incorporation of “safety switches” into the CAR construct, such as suicide genes that allow for the elimination of CAR T cells when necessary (173). In addition, to reduce the risk of prolonged immune activation, transient CAR expression systems using mRNA transfection or externally controllable CAR designs represent promising directions for therapeutic optimization (174).

As CAR T-cell therapy is progressively applied to autoimmune diseases, patients in this population often present with long-standing immune dysregulation and chronic inflammatory backgrounds, which may render them more susceptible to CAR T-related toxicities. Therefore, establishing a comprehensive toxicity management system is of critical importance. The integration of risk stratification and early intervention into a refined management framework holds promise for enhancing the overall safety of CAR T-cell therapy while maintaining its therapeutic efficacy.

## 7 Risk stratification and management for CAR T-cell therapy

For autoimmune diseases treated with CAR T-cell therapy, risk stratification—based on disease type, severity, immune status, and clinical characteristics—enables personalized treatment optimization to maximize efficacy. Since CAR T-cell therapy may trigger CRS and immune effector cell-associated neurotoxicity syndrome, risk stratification would help identify high-risk patients in advance and implement preventive measures to reduce adverse effects (175, 176). Furthermore, stratified management optimizes resource allocation by prioritizing patients with the highest predicted benefit, improving therapeutic success rates while enhancing safety through personalized risk mitigation (177).

For stratified management of CAR T-cell therapy in autoimmune diseases, patients can be categorized into B cell-mediated and T cell-mediated subtypes based on disease pathogenesis. This stratification approach enables precise treatment strategies tailored to distinct autoimmune subtypes, potentially enhancing efficacy while minimizing adverse effects. B-cell-mediated diseases, such as SLE and RA, are primarily driven by autoantibody production from B cells. Anti-CD19 CAR T cells can target and eliminate CD19-positive B cells, thereby reducing autoantibody production and inhibiting disease progression. In SLE patients, anti-CD19 CAR T-cell therapy induces profound B cell depletion, leading to reduced autoantibody titers and subsequent mitigation of tissue damage (81). Furthermore, CAAR T cells are specifically engineered to selectively target pathogenic B cell subsets while preserving the function of normal B cells, thereby reducing the side effects associated with long-term immunosuppressive drugs (124). For T cell-mediated diseases, such as T1D and multiple sclerosis, the pathology is mainly driven by autoreactive T cells. CAR Treg cells promote immune tolerance by suppressing autoreactive T cell activity, thereby attenuating inflammation and halting tissue damage (141).

In CAR T-cell therapy for autoimmune diseases, stratified management based on disease severity might help optimize treatment outcomes and reduce risks. For mild to moderate patients, low-dose CAR T-cell therapy is typically considered only when conventional immunomodulatory strategies prove ineffective or cause significant side effects. This approach could allow for gradual adjustment of the immune response, preventing overt immunosuppression (178). However, for severe or refractory patients who are unresponsive to standard therapies, CAR T-cell therapy might be used as a last resort to control systemic inflammation and organ damage by eliminating pathogenic immune cells. This treatment carries a higher risk of side effects, necessitating strict monitoring to prevent complications such as CRS (179, 180).

In CAR T-cell therapy for autoimmune diseases, individualized stratified management based on patient age and immune status would be crucial. Younger patients are typically able to tolerate higher doses of CAR T-cell therapy, which might result in more significant outcomes. In contrast, elderly patients, facing challenges such as immune senescence and comorbidities, would require lower doses of CAR T cells and strict monitoring for adverse effects to enhance safety (181, 182). Patients with normal immune function would be suited for standard treatment protocols, whereas those with impaired immune function, such as those on long-term immunosuppressive therapy or with immunodeficiency, might need tailored treatment adjustments to optimize efficacy and minimize side effects (183). All patients require continuous monitoring and adjustment of therapeutic strategies to ensure both safety and efficacy.

## 8 Preparatory conditioning chemotherapy regimens

The conditioning chemotherapy is a critical element in CAR T-cell therapy, designed to maximize the therapeutic potential of CAR T cells by promoting their proliferation, persistence, and efficacy within the patient’s body. It functions by depleting immunosuppressive Treg cells and myeloid-derived suppressor cells, as well as increasing the availability of key serum cytokines, such as IL-15 and IL-7, which further enhance CAR T cells activity and durability (177, 184). Additionally, conditioning chemotherapy reduces the existing lymphocytes in the patient’s body, creating a “space” for the infused CAR T cells to better proliferate and survive (185). In certain cases, it can also contribute to a reduction in the tumor burden, thereby enhancing the antitumor activity of CAR T cells (186).

The conditioning chemotherapy regimens currently used for autoimmune diseases are primarily adapted from those established for cancer treatment. In CAR T-cell therapy for cancer, common regimens include cyclophosphamide monotherapy, a combination of cyclophosphamide with fludarabine, or bendamustine monotherapy. Cyclophosphamide is generally reserved for specific cases or for patients who cannot tolerate more intensive regimens, and it is infrequently used as monotherapy (177). The cyclophosphamide-fludarabine combination chemotherapy regimen has been widely used in multiple clinical trials and has become the standard conditioning regimen for CAR T-cell therapy. The combined use of cyclophosphamide and fludarabine is believed to more effectively reduce immunosuppressive cells, thereby aiding in the expansion and long-term survival of CAR T cells. Compared to cyclophosphamide alone, the cyclophosphamide-fludarabine combination regimen has been shown to improve CAR T cells expansion and clinical outcomes (187, 188). Bendamustine, as an alternative option, is occasionally employed in patients who cannot tolerate the cyclophosphamide-fludarabine combination. Studies suggest that bendamustine can achieve comparable CAR T cells expansion but may reduce adverse effects, such as neutropenia, though its use is less common (189). In the context of autoimmune diseases, conditioning chemotherapy regimens for CAR T-cell therapy are often derived from those used in cancer but are adjusted in terms of drug selection and dosing. The cyclophosphamide-fludarabine combination regimen has shown promise in autoimmune disease patients, such as SLE, where it effectively reduces disease-driving autoreactive B cells and facilitates an immune system “reset” (81, 124). However, bendamustine is generally not recommended for use in autoimmune disease patients due to its potential for high toxicity, severe immunosuppression, and the lack of safety and efficacy data in this population (172, 175). Therefore, the cyclophosphamide-fludarabine combination is considered a safer and more effective conditioning regimen for autoimmune diseases and is more commonly recommended in clinical practice.

The conditioning regimen for CAR Treg therapy in autoimmune diseases differs from that of conventional CAR T-cell therapy, as CAR Treg cells are designed to enhance immune suppression to prevent autoimmune responses, rather than to eliminate B cells. Therefore, the selection of the conditioning regimen must be carefully considered to avoid impairing Treg cells’ function. Kanakry et al. showed that posttransplantation cyclophosphamide selectively preserved and promoted the reconstitution of Treg cells. This effect is primarily attributable to the high expression of aldehyde dehydrogenase (ALDH) in Treg cells, which metabolizes acrolein—a toxic metabolite of cyclophosphamide—thereby conferring resistance to cyclophosphamide in Treg cells (190). This characteristic suggests cyclophosphamide as an ideal preconditioning regimen for CAR-Treg therapy. Nevertheless, in autoimmune disease patients—many of whom are women of reproductive age—cyclophosphamide presents a concern due to its potential impact on fertility, necessitating careful evaluation (191, 192).

Bendamustine, due to its potent immunosuppressive effects, may impede the expansion and persistence of CAR Treg cells, potentially compromising therapeutic efficacy. Consequently, its application in CAR Treg therapy is generally limited (175). However, the necessity of pre-treatment chemotherapy for CAR Treg therapy in autoimmune diseases remains a topic of debate. Some studies suggest that pre-treatment chemotherapy may enhance the expansion and persistence of CAR Treg cells, while others raise concerns about introducing unnecessary toxicity and risk. As a result, pre-treatment chemotherapy is not universally required for all CAR Treg therapies, and decisions regarding its use should be made with caution, tailored to individual patient needs (193, 194).

## 9 Outlook: potential breakthroughs and future challenges of CAR T-cell therapy

The traditional CAR T-cell therapy process is complex and highly personalized, requiring the collection, genetic modification, and expansion of T cells from the patient, which results in long treatment cycles and may be accompanied by disease progression. In patients with autoimmune diseases undergoing immunosuppressive therapy, T-cell function is often suppressed, limiting the collection and expansion of autologous CAR T cells, thereby affecting the efficiency or yield of the final product, with a manufacturing failure rate of 2-10% (195). Moreover, the time-consuming processes of manufacturing, testing, and release, along with the logistical challenges of transporting cells between the treatment site and production facilities, pose significant risks, especially for patients with rapidly progressing or severe conditions. A pressing concern is that two major challenges currently hinder the clinical translation and large-scale trials of CAR T-cell therapy in autoimmune diseases: limited accessibility and high treatment costs. On the one hand, the therapy heavily relies on individualized cell manufacturing, complex genetic engineering platforms, and advanced cell therapy centers, making it difficult to implement routinely in most healthcare institutions. On the other hand, the high costs associated with production, quality control, and regulatory management result in overall treatment expenses far exceeding those of conventional therapies, imposing a significant burden on patients and healthcare systems. Therefore, reducing costs and improving accessibility have become critical bottlenecks that must be addressed to advance the broader application of CAR T-cell therapy. Therefore, the development of allogeneic CAR T technology offers a potential solution to overcome the limitations of traditional CAR T-cell therapies.

Allogeneic CAR T cells, also known as “off-the-shelf” CAR T cells (196, 197), are generated by genetically modifying T cells from healthy donors, enabling rapid therapeutic availability without the need to collect, process, and expand the patient’s own cells. Compared to the complex and individualized manufacturing process of autologous CAR T cells, allogeneic CAR T cells streamline the introduction of multiple cell modifications and achieve product standardization through donor selection and processing. This production approach significantly reduces costs through industrialization and large-scale manufacturing, allowing the generation of a substantial number of CAR T cells from a single donor. Furthermore, the production method for allogeneic CAR T cells enables the batch cryopreservation of T cells, ensuring treatments are readily available for patients. This approach not only saves resources and time but is particularly critical for patients with severe and acute conditions, as it meets their urgent need for timely therapeutic interventions (196). Additionally, the batch manufacturing of allogeneic CAR T cells facilitates the reuse of products when needed, providing a convenient option for retreatment. Most patients undergoing CAR T-cell therapy have been on long-term immunosuppressive treatments (such as corticosteroids, cyclophosphamide, tacrolimus, and rituximab) to control their condition, which can severely impair T cell quantity and function. As a result, the collection and expansion of autologous CAR T cells may be restricted, leading to the production of insufficient quantities or quality of autologous CAR T cells for treatment (196, 198). In such cases, using T cells from healthy donors to produce allogeneic CAR T cells becomes an ideal alternative. This approach not only provides a sufficient quantity of functionally robust T cells but also avoids the issue of suboptimal treatment outcomes due to the patient’s own T cells being functionally deficient or insufficient in number (199, 200). Compared to the personalized process of producing autologous CAR T cells, the production of allogeneic CAR T cells allows for scaling and standardization, significantly reducing costs, saving resources and time, and enabling coverage of a broader patient population (196). Moreover, allogeneic CAR T cells sourced from healthy donors can reduce the number of autoreactive cells in the patient’s body, thereby lowering the risk of relapse. For patients with autoimmune diseases, using allogeneic CAR T cells may also reduce treatment-related side effects, such as CRS (200, 201).

Although CAR T-cell therapy has demonstrated remarkable efficacy in the treatment of tumors and autoimmune diseases, it still faces challenges such as off-target effects, including non-specific attacks on healthy cells (175, 202). For instance, while eliminating pathogenic immune cells, CAR T cells may inadvertently attack normal immune cells, disrupting immune homeostasis or causing adverse effects such as CRS and organ damage. Off-target effects can also damage normal cells, reducing CAR T cell functionality and viability, thereby compromising therapeutic efficacy and increasing risks. The CRISPR/Cas9 gene-editing technology holds promise for enhancing the safety and efficacy of CAR T-cell therapy by mitigating off-target effects and non-specific actions.

Studies have shown (203–205) that directly delivering pre-assembled Cas9/gRNA ribonucleoprotein (RNP) complexes into cells allows Cas9 to rapidly exhibit activity. The rapid degradation of RNP significantly shortens Cas9’s duration of action, effectively reducing the probability of off-target cleavage while maintaining efficient and precise target editing. The combination of CRISPR/Cas9 gene-editing technology with RNP delivery strategy enables precise genome editing in CAR-T cells, thereby enhancing the specificity of target antigen recognition and reducing the risk of non-target antigen misrecognition that may lead to reduced efficacy or disease relapse. Additionally, this strategy facilitates multiplex gene editing and significantly reduces the risk of CRISPR off-target effects due to the transient activity characteristic of RNP delivery (206). *In vitro* experiments demonstrated that knocking out the SHP-1 gene in CD133-targeted CAR T cells significantly enhanced their cytolytic efficacy against CD133-positive glioblastoma cells. No Cas9 insertion or notable off-target effects were observed, confirming the potential of CRISPR/Cas9 technology to improve CAR T cell precision and reduce off-target effects (207). In a phase I open-label study targeting B-cell acute lymphoblastic leukemia, CRISPR/Cas9 technology was used to construct universal CD19/CD22 dual-targeted CAR T cells. The study enrolled six patients and employed high-fidelity CRISPR/Cas9 editing to knock out the TRAC and CD52 genes. Results demonstrated that the technology achieved high precision without observable off-target effects, while the treatment exhibited favorable safety and significant efficacy (208). By enhancing the specificity, persistence, and functional potency of CAR T cells, CRISPR/Cas9 gene-editing technology is expected to provide critical scientific evidence and technical support for the development of safe and effective CAR T-cell therapies for autoimmune diseases.

CAR T-cell therapy for autoimmune diseases is still in the early stages of clinical trials. While the results are promising, they are limited to a small number of patients, and long-term follow-up will reveal all potential safety issues. It is important to emphasize that these results are preliminary and require further investigation with longer durations and larger patient populations. A report by Mackensen et al. (81) on the use of anti-CD19 CAR T cells in 5 patients with refractory SLE showed that the patients tolerated the treatment well, with only mild CRS. Immature B cells reappeared on average 110 ± 32 days after CAR T cell infusion, and during a longer follow-up period (median (range) 8 (12) months post-CAR T cell administration), the patients remained in drug-free remission. Although this study report demonstrates that CAR T cells have the potential for long-term remission and good tolerability, it is important to consider that SLE patients may accumulate autoantibodies for several years before showing clinical symptoms. Therefore, even in cases of residual or re-emerging potential immune dysregulation, there could still be persistent clinical remission (209). Current studies have relatively short follow-up periods (typically ranging from a few months to about a year) and involve a small number of cases. To better evaluate the long-term effects and safety of CAR T-cell therapy, extended follow-up (five to ten years) is necessary to observe long-term remission rates, disease relapse rates, and potential delayed side effects. Special attention should be given to the chronic toxicity that CAR T-cell therapy may cause, including damage to internal organs, chronic inflammation, or fibrosis (175, 180). CAR T-cell therapy for autoimmune diseases requires further large-scale clinical studies to validate these preliminary results. Besides, long-term monitoring of patients’ immune system status is essential, with particular emphasis on the quantity, types, and functionality of regenerated B cells, as well as changes in T cell subsets, to assess the duration and quality of immune system reconstitution.

In autoimmune diseases, persistent inflammatory responses are compounded by pathological processes including autoimmune attacks, immune complex deposition, vasculitis, and tissue fibrosis, all of which can cause irreversible organ damage (210, 211). Even successful CAR T-cell therapy may fail to reverse established organ damage in autoimmune diseases, as demonstrated by SLE-related glomerulosclerosis, pulmonary fibrosis in systemic sclerosis, exocrine gland fibrosis in Sjögren’s syndrome, and demyelinating lesions in multiple sclerosis. This limitation highlights the critical importance of early intervention during the reversible disease phase. Initiating CAR T-cell therapy before irreversible structural changes occur can significantly improve treatment response rates and preserve long-term organ function.

Combined CAR T-cell therapy is emerging as an important approach for treating autoimmune diseases. In recent years, clinical research has explored the combined use of CAR T cells targeting different antigens, such as CD19 and BCMA, aiming to enhance therapeutic efficacy and overcome the limitations of single-target therapies. This strategy not only effectively eliminates various types of pathogenic cells but also has the potential to improve durability and breadth of treatment through multiple mechanisms. Preliminary clinical data demonstrate that BCMA-CD19 compound CAR T-cell therapy is safe and effective in patients with SLE (87). It not only induces medication-free remission but also significantly reduces pathogenic autoantibodies, showing potential for reducing relapse risk and achieving immune reconstitution. Additionally, combining CAR T with CAAR T offers new possibilities for future research. Anti-BCMA CAR T-cell therapy targets and eliminates pathogenic plasma cells, while CAAR T precisely target abnormal B cells responsible for autoimmune reactions. Their combination may generate synergistic effects, comprehensively clearing pathogenic cells and restoring B cell homeostasis. Although combination therapies show great promise, current research is still in its early stages. Future studies should focus on evaluating their safety, tolerability, and efficacy, as well as addressing potential immune responses and side effects induced by treatment. Overall, combined CAR T-cell therapy offer a novel therapeutic approach for autoimmune diseases, warranting further exploration and clinical validation.

It is noteworthy that most current CAR T-cell therapies primarily target B cells, particularly CD19^+^ B cells and their pathogenic plasma cell derivatives. However, the pathogenesis of many autoimmune diseases is not solely B-cell driven but heavily dependent on T cells—especially CD4^+^ helper T cells reactive to the same autoantigens. The generation of long-lived plasma cells typically requires T-cell help, and the autoantibodies they secrete are antigen-driven. Therefore, even if pathogenic B-cell populations are eliminated, residual autoreactive CD4^+^ T cells may reinitiate the activation of newly regenerated B cells, leading to the re-emergence of pathogenic autoantibodies and increasing the risk of disease relapse. To address this concern, future CAR T-cell strategies may benefit from incorporating approaches that also target autoreactive T cells—such as the development of CAR T cells specifically directed against pathogenic T-cell subsets, or combinatorial strategies involving CAR-Tregs to promote immune tolerance. Furthermore, longitudinal monitoring of CD4^+^ T-cell subsets and their antigen specificity following therapy will be essential to assess whether true immune reprogramming has been achieved. Therefore, optimizing CAR T-cell therapies for autoimmune diseases will require advances in target precision, understanding of autoantigen-driven responses, and elimination of immunological memory to improve long-term efficacy and minimize relapse risk.

To ensure the standardization and normalization of the treatment process, and to enhance patient safety and treatment efficacy, it is essential to establish global consensus guidelines for CAR T-cell therapy in autoimmune diseases. Given the complexity of CAR T-cell therapy for autoimmune conditions, which involves extensive multidisciplinary knowledge, detailed guidelines are needed to direct clinical practice. This will help standardize treatment procedures and reduce variability. Moreover, it will provide clear standards for treatment and side-effect management, ensuring patients receive optimal care while minimizing treatment-related risks. By developing consensus guidelines, multidisciplinary collaboration can be promoted, evaluation standards for treatment efficacy and side effects can be unified, and treatment strategies can be optimized, thereby improving overall safety and effectiveness. Furthermore, such guidelines can advance scientific research, help formulate more scientific and forward-looking treatment protocols, and enhance patient and public understanding and acceptance of the treatment options.

In conclusion, CAR T-cell therapy represents a novel approach to the treatment of autoimmune diseases, offering the potential for durable immune reconstitution and even a cure by specifically eliminating autoreactive B cells. Its remarkable efficacy has been demonstrated in refractory autoimmune diseases, such as SLE. Future innovations, including mRNA-engineered CAR T, “off-the-shelf” allogeneic CAR T-cell therapies, and combination CAR T-cell therapy are expected to enhance therapeutic outcomes, reduce toxicity, and lower costs. Additionally, risk stratification strategies will optimize personalized treatment, minimize adverse effects, and improve clinical safety and efficacy. Together, these advances position CAR T-cell therapy as a transformative frontier in autoimmune disease management, with the potential to achieve sustained remission and cures.
